# Editorial: Experimental hernia research from bench to bedside and translational perspectives, volume II

**DOI:** 10.3389/fsurg.2025.1699360

**Published:** 2025-10-03

**Authors:** René H. Fortelny, Barbora East

**Affiliations:** 1 Department of General Surgery, Sigmund Freud Private University Vienna, Vienna, Austria; 2 General Surgery, Motol University Hospital, Prague, Czechia

**Keywords:** hernia research, translational research, abdominal wall hernia, inguinal hernia, hernia repair

Hernia research has undergone remarkable transformation in recent decades, moving beyond purely technical refinements of surgical repair toward a multidisciplinary field encompassing molecular biology, biomaterials science, regenerative medicine, and patient-centered outcomes. This evolution reflects a broader trend in surgery: the recognition that durable and individualized repair strategies require not only surgical expertise but also translational insights that bridge basic science with clinical application.

Experimental hernia research today addresses fundamental biological mechanisms that underlie abdominal wall failure, including altered collagen metabolism, impaired wound healing, and biomechanical tissue weakness. Parallel advances in mesh design, resorbable scaffolds, and biologically active materials have provided new opportunities to modulate the host response and reduce long-term complications such as chronic pain, fibrosis, and recurrence. The growing availability of *in vitro* models, animal studies, and computational simulations has further enhanced our ability to study complex interactions at the cellular and tissue levels.

Translating these findings into clinical practice remains both a challenge and a priority. The concept of “bench to bedside” in hernia surgery emphasizes not only the safe introduction of innovative techniques and materials, but also the necessity of robust clinical trials, registries, and long-term follow-up. Equally important is the reverse translation—bringing clinical observations back to the laboratory to refine hypotheses and guide future research.

A translational perspective also highlights the importance of interdisciplinary collaboration. Surgeons, basic scientists, material engineers, and industry partners must work closely to ensure that novel discoveries are effectively implemented and evaluated. At the same time, patient-reported outcomes and health economic considerations are essential to define the true value of innovation in hernia care.

In this context, experimental hernia research represents a unique opportunity to redefine the future of abdominal wall surgery. By integrating mechanistic understanding with clinical relevance, and by fostering continuous exchange between laboratory and bedside, the field can move toward safer, more effective, and more personalized repair strategies.

